# Psychosocial resilience surrounding age-typical losses among older adults in Sweden: group-based trajectories over a 25-year-period

**DOI:** 10.3389/fpubh.2024.1434439

**Published:** 2024-10-24

**Authors:** Neda Agahi, Erika Augustsson, Christine McGarrigle, Tine Rostgaard, Johan Fritzell

**Affiliations:** ^1^Aging Research Center, Department of Neurobiology, Care Sciences and Society, Karolinska Institutet and Stockholm University, Stockholm, Sweden; ^2^School of Medicine, Trinity College Dublin, The University of Dublin, Dublin, Ireland; ^3^Department of Social Sciences and Business, Roskilde University, Roskilde, Denmark; ^4^Department of Social Work, Stockholm University, Stockholm, Sweden

**Keywords:** life satisfaction, widowhood, functional loss, resilience, anticipatory grief

## Abstract

**Introduction:**

Resilience is an important but often neglected aspect of healthy or successful aging. This study used a new approach for modelling psychosocial resilience to investigate responses when faced with age-typical losses. Rather than viewing resilience as a trait, we modelled trajectories of life satisfaction when faced with partner or functional loss in later life to identify resilient trajectories. Then, we examined characteristics associated with belonging to resilient trajectories.

**Method:**

We analyzed longitudinal data from the individually linked Swedish LNU and SWEOLD surveys collected repeatedly between 1991 and 2021 (*N* = 450). A group-based trajectory modelling technique was used to obtain trajectories of life satisfaction over time, centered around the first identified loss. Identified losses (widowhood, loss of mobility and loss of vision), occurred between ages 60 and 98.

**Results:**

Four trajectories of life satisfaction surrounding loss were identified, labelled Declining (46%), Non-recovering (24%), Recovering (16%), and Bouncing back (14%). The latter two were categorized as resilient. Individuals in resilient trajectories were more likely to be younger, engaged more with their friends after the loss, reported fewer mental health problems both before and after the loss, and had a higher sense of control before the loss.

**Discussion:**

Several interesting patterns of long-term change in life satisfaction were found in response to loss. Contrasting earlier findings, only about one-third of the sample was categorized as resilient. The two resilient trajectories were characterized by either quick adaptation or a slower adaptive process. Increasing or maintaining social networks, particularly friendships, after an age-typical loss may promote resilience.

## 1 Introduction

In light of population aging, identifying ways to strengthen individuals’ social and personal resources to foster resilience and enhance wellbeing has become increasingly important. Although very central to healthy or successful aging, the concept of resilience has largely been neglected in this context. In recent years, however, it has gained increasing interest in the empirical and theoretical investigations of later life (1, 2, 52). Resilience can be defined as “the process and outcome of successfully adapting to difficult or challenging life experiences” (3), and is considered a dynamic process encompassing positive adaptation when faced with significant adversity (4, 53, 54). In this study, we explore psychosocial resilience when faced with critical life event losses that become more common at older ages: loss of partner and loss of physical functions. Drawing on Rutter (5) and Masten (6), we view psychosocial resilience as the dynamic set of processes, involving both inherent individual traits and resources and social support, that lead to an adaptive response to challenging life experiences. To do this, we move beyond cross-sectional data and try to capture adaptive responses over time rather than individual traits. We use high-quality longitudinal survey data of older people in Sweden, followed up multiple times over 30 years, and ask the following overarching questions: what characterizes resilient trajectories (if any) in older age and what are the individual, social and (mental) health characteristics that may enable older individuals to adapt to adverse events?

Different stressors and adverse events may, and do, occur over the life course. In later life, these typically include the loss of a partner or a decline in physical health and functioning (7, 8). In the US, about 40 percent of adults aged 65 and older have experienced the death of a partner (9). Percentages are similar in European countries [e.g., (10)]. Losing physical function, such as walking ability and vision, are other common experiences at older ages. Experiencing such losses often negatively affects the person’s mental well-being and may result in a lower quality of life (11–14). For some, the decline in mental well-being remains, while others can adapt to their new circumstances and return to their former state. The concept of resilience attempts to capture this adaptation following adversity (4, 55).

The concept of resilience has been used in several disciplines, referring to the adaptation or elasticity of ecosystems, materials and human beings when put under stress or shocks. Research on human resilience has been defined and measured in different ways [for an overview, see, e.g., (15, 16)]. Many studies have used resilience scales to measure an individual’s inherent level of resilience, thereby viewing resilience as an individual trait [e.g., (17–19)]. However, resilience is increasingly viewed as dynamic and shaped by the social context, in response to a stressor. In line with this view, other studies have investigated individuals’ ability to adapt or “bounce back” after adverse events [e.g., (20, 21)]. This adaptation takes place in interaction with the environment, modelled by Sandler et al. (22) as “contextual resilience.” Their idea is that when an adverse event takes place, it disrupts the individual’s roles and relation to the environment (e.g., of being a spouse, someone people can rely on for help, an independent individual etc.), which poses a threat to the individual’s level of well-being. Over time the individual may adapt to the new situation, with the use of both individual and social resources (22). Our approach is in line with this view. We consider resilience as a long-term process surrounding age-typical loss and operationalize it as the return to pre-loss levels of well-being. The adaptation process may take various forms and differ in time and effort needed; previous studies have found large heterogeneity in how individuals respond to losses such as bereavement, divorce, and disability [e.g., (11, 23)].

The measure by which we model psychosocial resilience is life satisfaction. Theoretically, one often distinguishes between affective well-being and life satisfaction as two forms of subjective well-being or happiness (24, 56). Life satisfaction is a broader concept that leaves more room for the individual agent to decide what is important in life. Empirically, many studies use life satisfaction as the operational concept [e.g., (23, 25, 26)]. Life satisfaction also exhibits greater responsiveness to change after negative events compared to positive affect (27). Considering resilience as the adaptation following age-related losses, trajectories in life satisfaction can serve as a way to measure resilience. This aligns with the concept of psychosocial resilience in older age, particularly in relation to disease accumulation and functional loss (28). While in general, life satisfaction is believed to remain relatively stable or even improve as people enter older ages, this does not apply uniformly to all individuals and all circumstances (29). Indeed, findings suggest that life satisfaction is affected more among older adults, particularly the oldest old (85+), than younger age groups with the onset of disability (11).

As mentioned above, previous research on resilience suggests that it is largely a product of individual and environmental factors combined (15, 22, 30). These factors, or resources, can either improve the individual’s own ability to face the stressor, help the individual meet the demands posed by the stressor, or protect against the negative effects of the stressor, thereby resulting in a resilient response (15). On the individual level, factors such as health and more internal factors such as coping styles or the ability to solve problems, manage emotions or accept own circumstances have also been linked to resilience. On the environmental level, access to and quality of social resources such as social relations and support have been identified as important aspects of resilience [e.g., (3, 15, 31)]. We will focus mostly on social resources, but to some extent also on people’s individual psychological resources, such as mental health and internal abilities (e.g., perceiving that life is comprehensible and that one is in control of one’s life). Studies about social factors in relation to resilience in older adults are scarce, especially in the general population. Overall, social resources such as being married, having more social support, and a larger social network are positively related to resilience in general population samples (32), and people with higher social participation before disability onset seem to respond better to the functional loss (11). Studies on older age groups also found that social networks were important; more social support and higher contact frequency with friends and family were associated with resilience (21, 33–35). A meta-synthesis of qualitative studies identified social connections as central to resilience for older adults, as well as various psychological resources such as meaningfulness, grit, and a positive outlook on life (36). Most of these studies, however, were conducted on cross-sectional data with resilience modelled as an individual trait. We will further investigate how these factors are associated with long-term trajectories of life satisfaction and resilience.

Despite the fact that late life is a period of many changes and transitions, most studies of changes in life satisfaction or wellbeing surrounding critical life events have not focused specifically on older adults. It could be that because certain losses (such as loss of partner and physical function) are more or less expected and perhaps considered a normal part of the later life-course, it is easier for older individuals to accept and adapt (26), perhaps especially in the case of bereavement if the loss has been preceded by strenuous informal caregiving and therefore comes as a relief. It can also be that because of the many losses and difficulties that occur in later life for some people, the available resources to withstand further losses is limited (11).

To fill some of these research gaps, this study explores long-term trajectories of life satisfaction and resilience processes surrounding age-typical losses (loss of a partner or onset of functional limitations), happening after age 60. We follow a national sample of older individuals in Sweden over a long period and analyze latent trajectories of life satisfaction around the first occurrence of such losses. The primary aim is to identify trajectories that suggest a resilient response to these losses. A secondary aim is to investigate individual, social, and mental health characteristics that are associated with membership of resilient trajectories.

## 2 Data and methods

### 2.1 Data

This study used individually linked nationally representative survey data from the Swedish Level of Living Survey (LNU) (37) and the Swedish Panel Study of Living Conditions of the Oldest Old (SWEOLD) (57). The sample of the LNU survey is randomly drawn from the Swedish population aged 15 (in later waves 18) to 75 years in Sweden (*n* = 6,000–9,000) and was initiated in 1968, with follow-ups in 1974, 1981, 1991, 2000, 2010 and 2021. The same individuals are contacted each time, as well as an additional sample of younger persons and immigrants to keep the sample nationally representative. Individuals who pass the upper age limit of 75 years are followed up in the SWEOLD study. SWEOLD has been carried out in 1992, 2002, 2004, 2011, 2014, and 2021. The data collections in 2004 and 2014 had lower age limits of 69 and 70 years, respectively. Both LNU and SWEOLD have used face-to-face interviews as the main data collection mode, until the most recent data collection where telephone interviews were conducted because of the Covid-19 pandemic. Telephone interviews were also used in 2004 and 2014. To increase response rates and facilitate participation, paper questionnaires and mixed or indirect interviews with a family member or care staff were also used (57). Response rates are generally high, ranging from 60.9 to 79.1% in the LNU surveys included here (1991–2010), and 64 to 85% in the included SWEOLD surveys (2002–2021).

### 2.2 Study sample

**Figure 1:**
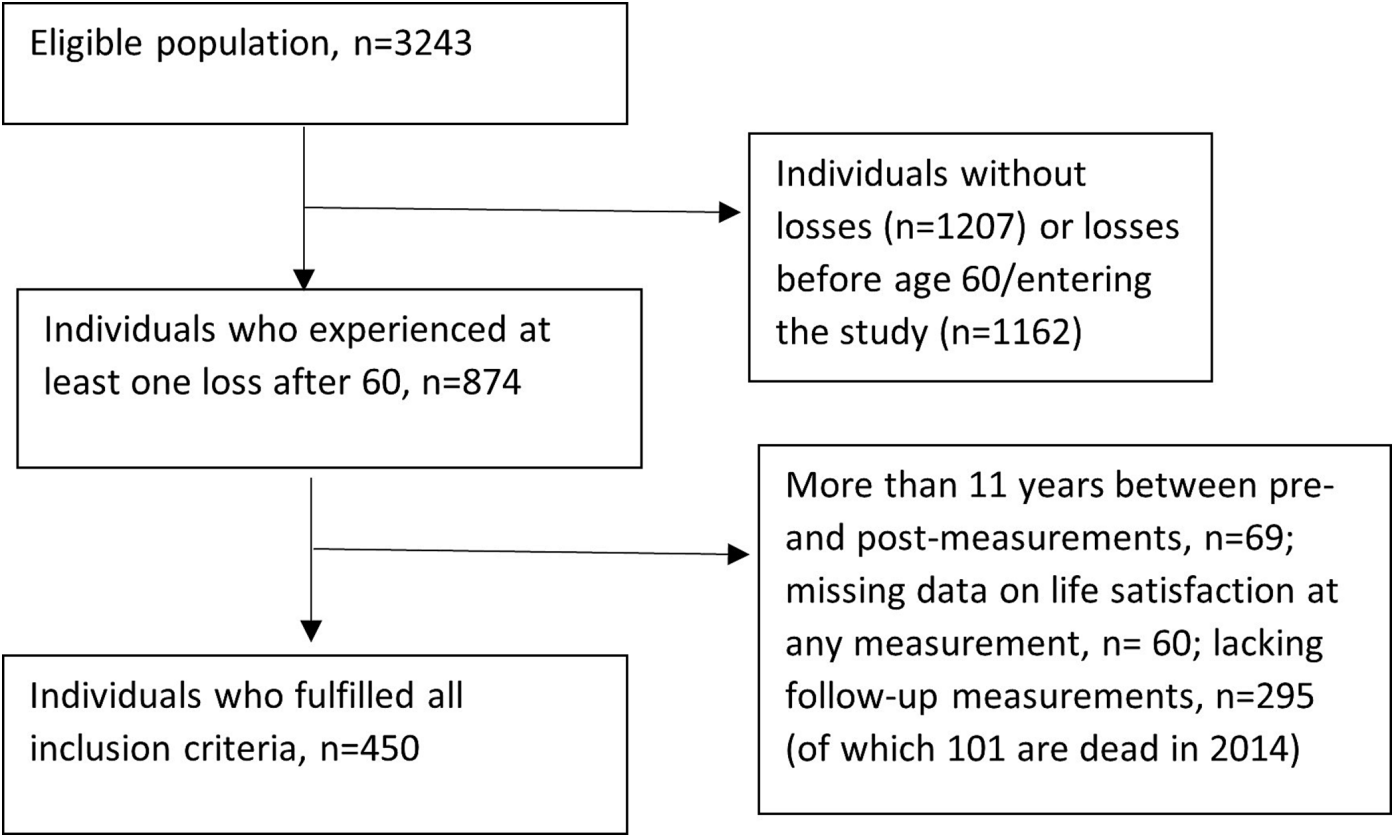
Flow-chart for study sample.

The current study used data from all waves of both LNU and SWEOLD, from 1991 and onwards. All interviewed individuals without the experience of loss of partner, walking ability and vision at the first observation point (more details below) were eligible to be part of the study sample. From this sample, we prospectively identified individuals with at least one loss. Data were centered around the first identified loss after the age of 60. Individuals with intervals between the pre-and post-measurements of more than 11 years were excluded from the analyses, as were persons with less than one measurement before and two after the loss. The analytical sample consisted of 450 individuals, see flowchart in Figure 1. In summary, the persons included in the sample participated in at least three waves; one measurement before the loss, one measurement within 11 years of the previous measurement and a recorded loss in between, and one additional follow-up measurement. However, they could have more measurements, up to 10 years before the event and 15 years after the event. Average follow-up times are reported in Table 1. For a visualization of a randomly selected sample of the data, see Supplementary Figure S1.

**Table 1:** Characteristics of the total sample and the four trajectory groups.

	Total	Non-resilient trajectories		Resilient trajectories	
		Declining	Non-recovering	Recovering	Bouncing back
	*n* = 450	*n* = 208	*n* = 110	*n* = 70	*n* = 62
Age-typical losses					
First loss(es) after 60 (%)					
Loss of partner	41.8	39.4	40.9	55.7	35.5
Loss of walking ability	44.2	50.0	39.1	42.9	35.5
Loss of vision	23.6	24.0	30.0	8.6	27.4
Total number of losses	1.5	1.6	1.3	1.4	1.5
Range	1–3	1–3	1–3	1–3	1–3
Standard deviation	0.64	0.69	0.61	0.61	0.53
Mean years between pre-and post-loss measurement and the loss	4.1	3.9	4.4	4.2	4.2
Standard deviation	1.34	1.39	1.19	1.37	1.24
Mean years between post-loss measurement and follow-up	3.9	3.9	3.7	3.7	4.1
Standard deviation	2.0	1.9	1.8	2.0	2.3
Demographics					
Women (%)	62.7	58.2	66.4	68.6	64.5
Mean age at time of loss	73.0	73.0	73.2	72.4	71.0
Standard deviation	6.89	7.70	7.36	6.38	6.72
Range	60–90	60–90	60–88	60–88	60–87
Beyond compulsory education (%)	54.0	48.0	60.0	50.0	67.7
Married before the loss (%)	85.78	80.29	90	90	91.94
Has children (%)	90.0	92.3	80.9	94.3	93.6
Social characteristics					
Meeting friends (mean)					
Before	2.6	2.5	2.7	2.6	2.7
After	2.4	2.1	2.3	2.7	2.9
Long after	2.2	2.0	2.2	2.2	2.7
Meeting relatives (mean)					
Before	2.5	2.5	2.5	2.6	2.6
After	2.1	2.0	2.2	2.2	2.4
Long after	1.8	1.7	1.9	1.9	2.0
Has social support (%)					
Before	94.6	92.7	98.1	91.3	98.4
After	91.7	91.0	94.4	89.6	91.7
Long after	95.3	94.0	93.9	100.0	96.7
Lives alone (%)					
Before	15.1	19.7	13.6	10.0	8.1
After	54.7	56. 5	46.4	64.3	53.2
Long after	58.2	56.7	53.6	68.6	59.7
Sense of control before the loss (mean)					
Do not understand things that happen	1.3	1.4	1.7	1.2	1.3
Not in control of one’s life	1.4	1.5	1.5	1.2	1.3
Mental Health					
Anxiety (%)					
Before	16.9	23.1	16.4	5.7	9.7
After	22.2	29.6	16.5	21.4	8.2
Long after	26.4	30.7	28.7	18.8	16.4
Depression (%)					
Before	5.3	8.2	3.6	4.3	0
After	14.0	19.6	8.3	15.7	3.3
Long after	14.7	20.2	12.8	8.7	6.6

Percentages and mean values. *N* = 450. Before = 1 to 5.5 years before the loss; After = 1 to 5.5 years after the loss; Long after = 3 to 15 years after the loss.

### 2.3 Main variables

*Age-typical losses* included in the analyses were the loss of a partner and loss of functional ability, measured as walking ability and vision. *Widowhood* was indicated by the respondent in a question about marital status. Participants who reported being married or cohabiting in one data collection and widowed in a subsequent one were coded as having lost a partner. Similarly, loss of walking ability was identified through a question asking whether the respondent was able to walk 100 meters without difficulties, if they responded yes in one data collection and no in a subsequent one, this was an indication of a loss of walking ability. Finally, loss of vision was measured as having severe eye problems or eye disease which is not aided by glasses in one data collection but not in a preceding one. These questions were available in all waves. As mentioned above, time was centered around the first time a loss was recorded after age 60.

*Life satisfaction* was measured with the question “In general, do you think that your situation is very good (5), rather good, neither good nor bad, rather bad, or very bad (1)?” up to and including 2010, thereafter the question was phrased as “Thinking about your own life and personal circumstances, how satisfied are you with your life as a whole (today)?” with the options of a scale running from 0 (completely dissatisfied) to 10 (completely satisfied). The scale was recoded into 0.5-step increments running between 0.5 and 5, to resemble the 5-point scale. One-item life satisfaction measurements have been found to be valid and very similar to multiple-item measurements (38).

### 2.4 Demographics

*Education level* was self-reported and coded into compulsory schooling (between 6 and 9 years of schooling in these birth cohorts) and beyond compulsory schooling.

Being *married before the loss* was assessed at the time-point before the loss. Self-reported civil status was measured as married/cohabiting, single, divorced or widowed, dichotomized as married/cohabiting vs. not. Individuals who were widowed prior to the event were excluded from the analyses.

Two measures of social relationships were collected: *meeting friends* and *meeting relatives*. They were measured with two items each: “How often do you visit friends/relatives at their home?” and “How often do your friends/relatives visit you in your home?.” Response options were “no” (0), “yes, sometimes” (1), and “yes, often” (2). These two items were summed into a score of 0–4 for friends and relatives separately.

*Social support* was measured with the question: “Sometimes you need help and support from someone. Do you have a relative or close friend who can help you if you need someone to talk to about your personal problems?.” Response options were yes and no.

We also included two variables that describe people’s *sense of control*: “Do you usually feel that things that happen to you in your daily life are hard to understand?” and “Do you usually feel that you are in control of your own life?.” Response options were “no,” “yes, sometimes,” and “yes, often.”

*Mental health* was measured with two items: self-reported depression/deep sadness and self-reported anxiety/worry/nervousness experienced during the past 12 months. Responses were “no,” “yes, mild,” and “yes, severe,” dichotomized into no and yes.

### 2.5 Statistical analyses

Analyses were conducted in two steps. First, a group-based trajectory modelling technique was used to obtain trajectories of life satisfaction over time surrounding loss of partner or physical function. We used latent class growth analyses (LCGA), an exploratory, data-driven analysis based on finite mixture modelling which derives patterns based on clustering through a maximum likelihood function (39). The choice of this method was based on our aim to distinguish individuals following distinctive trajectories over the time surrounding a loss (40). While LCGA is a straightforward, easily interpreted, commonly used, and computationally less demanding method, it has been critiqued for its inherent assumption that individuals within each class (in our case each trajectory) do not differ from each other with regard to intercept and slope, thereby overfitting the data and finding more classes than there are in the data [e.g., (25, 41)]. As described below, we have used several strategies to allow for more flexible modelling and trajectory shapes, and when choosing the number of groups/trajectories that best described the data.

First, data was centered around the event of an age-typical loss. As mentioned above, the loss was identified if a partner/physical function was present in one data collection but lost in the subsequent one. As the exact time of the loss is not available in the data, we set the time for the loss/event (i.e., time = 0, around which the data was centered) in the exact middle of the two surrounding data points. In other words, for individuals with an interval of 4 years between pre-and post-measurements, the event was assumed to take place 2 years after the pre-measurement and 2 years before the post-measurement. Participants required at least one measurement before and two after the age-typical loss to be able to model non-linear trajectory shapes, additional measurements were included for those who were part of more than three waves. We used a censored normal probability distribution. Models with up to five groups, with options of linear, quadratic, and cubic trajectories were tested using an algorithm, and the 10 best-fit models using the Bayesian Information Criterion (BIC) were further investigated. For best model fit we used BIC, average posterior probability >0.7, odds of correct classification >5, entropy >0.7, group size bigger than 5 per cent, and no bigger difference between predicted and actual group size than 4 per cent [criteria based on (39), and (42)]. A five-group solution had the best BIC fit, however while lower BIC was preferred, we considered all model fit criteria when selecting models, therefore we chose a solution with four groups that fit model criteria and theoretical assumptions of trajectory patterns better. The 4-group solution produced similar trajectories to the 5-group solution with the highest BIC values. The trajectories were then described visually. All individuals were categorized as belonging to the trajectory with the highest posterior probability. Descriptive statistics of various social and health characteristics across the four trajectory groups were then investigated.

Second, a binary variable that classified the trajectories as resilient or not was created, grouping resilient groups (Recovering and Bouncing back) together. This was used as the outcome in logistic regressions to estimate the likelihood of belonging to resilient and non-resilient trajectory groups based on sociodemographic and social characteristics.

## 3 Results

**Figure 2:**
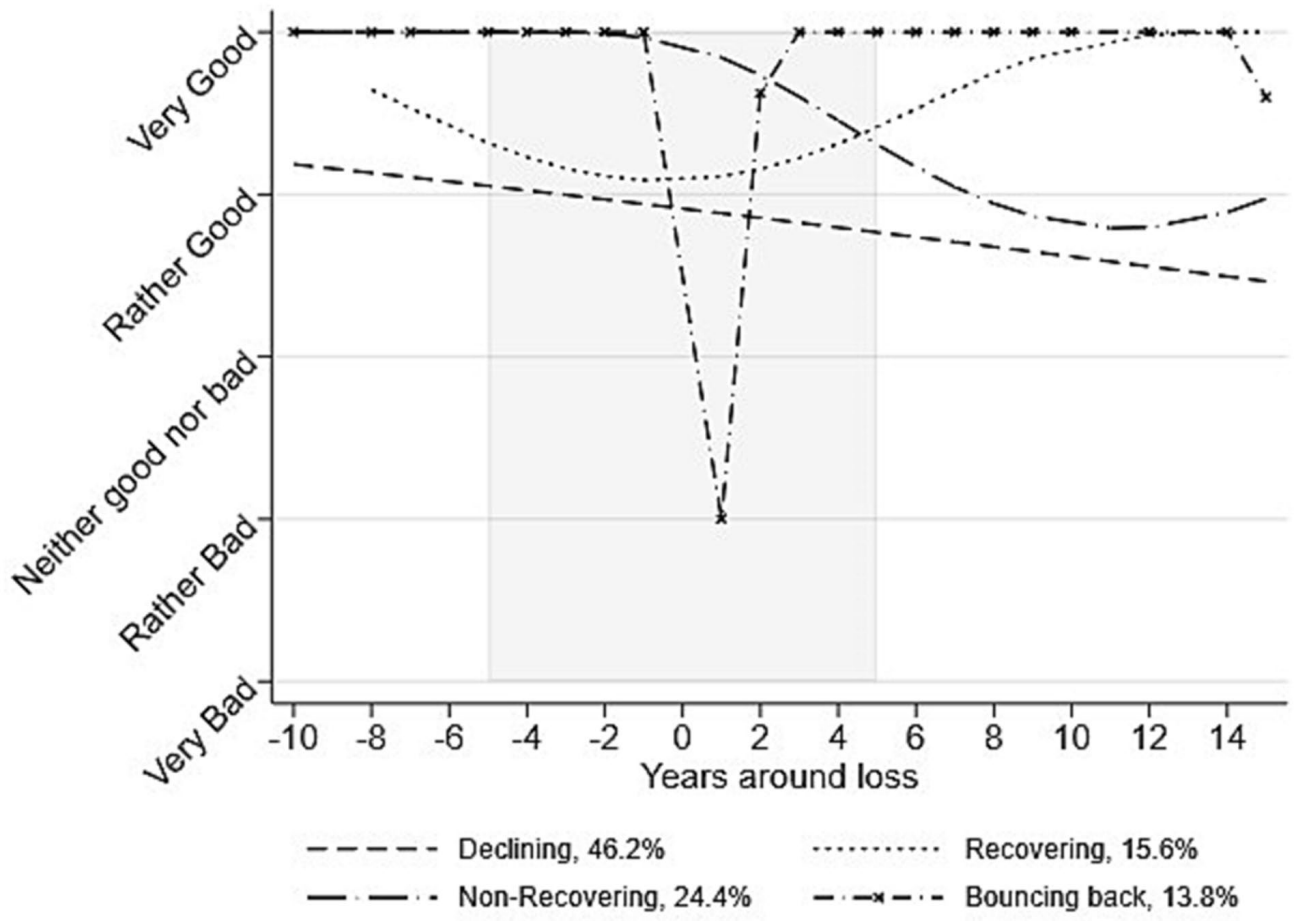
Trajectories of life satisfaction surrounding age-typical losses. The x-axis shows years before and after the first identified loss, which is assumed to happen at time 0. The y-axis shows life satisfaction on a 5-point scale. The grayed area indicates the time span in which the loss can have taken place.

Figure 2 shows the trajectories of life satisfaction before and after age-typical losses in later life. The solution with four groups; one linear, two quadratic, and one cubic trajectory, best described the data, see supplementary materials for model fit indices (Supplementary Table S1). Four trajectories of life satisfaction were identified. The “Declining” trajectory, estimated to include 46.2% of the sample (*n* = 208), was the most common trajectory. It started a bit lower than the other trajectories and showed a stable gradual decline over the whole time period, seemingly unaffected by the loss. In the “Non-recovering” trajectory, including 24.4% of the sample (*n* = 110), individuals had high levels of life satisfaction prior to the loss and then a clear decline with no recovery in connection with the loss. In the “Recovering” trajectory, including 15.6% of the sample (*n* = 70), individuals showed decreasing life satisfaction before and surrounding the loss but then gradually increasing back to high levels. Finally, in the “Bouncing back” trajectory, including 13.8% of the sample (*n* = 62), individuals had a sharp drop in life satisfaction immediately surrounding the loss but high levels of life satisfaction before and after this drop. These last two trajectories are considered resilient.

Table 1 shows the characteristics of people in the sample as a whole as well as in the four trajectory groups. In the total sample, the most common loss was the loss of walking ability, closely followed by loss of a partner (44 and 42 percent respectively), and least common was loss of vision (24 percent). Women made up 63 percent of the sample, mean age at first loss was 73 years, but age spread was wide. More than half the sample had higher than compulsory education (5–8 years in these cohorts), most were married before the loss and had children. Engagement with both friends and relatives was quite high before the loss and then decreased over time. Scores for not understanding things that happen or not feeling in control of one’s life were quite low (only measured before the loss). Mental health problems, both anxiety and depression, increased over time from before the loss and throughout the follow-up period.

In the largest trajectory group, the “Declining” group, a small majority were women, but the proportion of men was larger compared to the other trajectory groups. Among all men, about 50 percent were in the Declining group, whereas only about 40 percent of all women were in this group (not shown). The most common loss in the Declining group was the loss of walking ability followed by the loss of a partner, and individuals in this group experienced the highest number of losses. Compared to the other groups, social engagement with friends and relatives was slightly lower. Mental health problems were highest in this group throughout the period with notable increases in depression.

In the “Non-recovering” group, two-thirds were women, and mean age was higher than in the two resilient groups. The distribution between the three kinds of losses was quite even in this group, and they had the lowest number of losses. This group had the highest percentage of individuals without children. The education level was higher than in the “Declining” group, with 60 percent having beyond compulsory education. Social networks, both with friends and relatives, decreased during the follow-up period as did social support. Mental health problems increased over time. This group had the highest mean values of not understanding things that happen or not feeling in control of one’s life.

The “Recovering” group had the highest proportion of women. A majority of this group experienced the loss of a partner, followed by the loss of walking ability. Meanwhile, a very small percentage of this group lost their vision. Mental health problems were low before the loss, increased post-loss, to then decrease again, though anxiety remained more common. This group had the lowest mean values of not understanding things that happen or not feeling in control of one’s life.

Finally, the “Bouncing back” group consisted of about 65 percent women, had the lowest mean age at the time of loss (71 years), and the highest education level. They also had the lowest proportion of loss of partner and walking ability compared to the other groups. Furthermore, this group had the highest overall level of social engagement with both friends and relatives and the lowest levels of mental health problems both before and after the loss.

In Table 2, the two resilient groups were combined and compared to the two non-resilient groups. Table 2 shows the individual, social and health characteristics associated with membership of resilient trajectories. Results show that individuals in the resilient trajectories are different in some domains. They are more likely to be younger when they experience their first loss (aged 65–74 compared to the reference group 75–84), more likely to have children, to meet with others (particularly friends) more often after the loss, and finally to perceive the world as more understandable and feel that they are in control of their life (before the loss). Those with lower mental health problems throughout the study period, particularly anxiety, also had a higher probability of being in the resilient trajectory.

**Table 2:** Characteristics of the resilient trajectory groups.

	Crude model	95% CI	Age-adjusted	95% CI
Demographics				
Women	5.0	−3.6; 13.6	4.8	−3.8; 13.3
Higher Education	5.1	−3.3; 13.5	3.9	−4.6; 12.4
Age at the first event (continuous)	−0.6	−1.1; 0.02		
Age category at first event				
60–64	0.0	−1.2; 1.3		
65–74	**13.4**	**4.2; 22.7**		
75–84	(ref)			
80+	−8.1	−25.6; 9.5		
Social characteristics				
Has children	**12.8**	**0.8; 24.9**	12.1	−0.2; 24.4
Meeting friends				
Before	1.2	−2.7; 5.0	1.0	−2.8; 4.8
After	**8.6**	**5.2; 11.9**	**8.3**	**4.8; 11.8**
Long after	**4.8**	**1.6; 7.9**	**4.2**	**0.95; 7.5**
Meeting relatives				
Before	1.6	−2.3; 5.5	1.2	−2.7; 5.2
After	**3.7**	**0.1; 7.3**	**3.2**	**−0.5; 6.9**
Long after	2.8	−0.7; 6.4	2.4	−1.2; 6.0
Sense of control before the loss				
Do not understand things that happen	**−12.6**	**−21.3; −3.8**	**−12.6**	**−21.3; −3.9**
Not in control of one’s life	**−8.2**	**−15.1; −1.3**	**−8.2**	**−15.0; −1.3**
Mental health				
Anxiety				
Before	**−23.4**	**−37.2; −9.8**	**−22.2**	**−36.0; −8.4**
After	**−12.7**	**−23.6; −1.8**	**−11.1**	**−22.2; −0.08**
Long after	**−14.1**	**−24.3; −3.9**	**−13.7**	**−23.8; −3.5**
Depression				
Before	−22.9	−47.9; 2.2	−23.1	−48.0; 1.8
After	−10.8	−24.1; 2.5	−9.5	−22.9; 3.9
Long after	**−19.3**	**−33.4; −5.1**	**−18.2**	**−32.4; −4.0**

Average Marginal Effects with 95% confidence intervals (CI). *N* = 450.Before = 1 to 5.5 years before the loss; After = 1 to 5.5 years after the loss; Long after = 3 to 15 years after the loss. Statistically significant estimates marked in bold.

## 4 Discussion

This study investigated long-term trajectories of resilience in older age by analyzing life satisfaction surrounding age-typical losses in later life, and the individual, social, and mental health characteristics associated with these trajectories. To our knowledge, this is the first study that investigates resilience in the older population with this modelling approach and over such a long period. This approach adds to the literature by focusing on age-typical losses and by investigating changes over a more extended time than previous research. Results showed four main trajectories of life satisfaction surrounding age-typical losses: Declining (46%), Non-recovering (24%), Recovering (16%), and Bouncing back (14%). The two latter trajectories were categorized as resilient, but with different responses to the loss. While the adaptation process was quick in the Bouncing back group, the Recovering group had a slower adaptive process but eventually returned to high levels of life satisfaction. About a third of the study sample had a resilient response when faced with age-typical losses. This contrasts with earlier findings showing that most individuals respond to loss with resilience [e.g., (30)], perhaps because we investigated older age groups. Lower occurrence of resilience is however in line with other findings suggesting that resilience in response to loss or trauma may have been overestimated in earlier studies (26, 43). Moreover, we found that individuals in the resilient trajectories were more likely to be younger, engage more with their social network after the loss, particularly their friends, report fewer mental health problems both before and after the loss, and perceive life as more understandable and controllable before the loss.

Several interesting patterns emerged in the trajectories. In the “Recovering” trajectory, the decrease in life satisfaction started prior to the loss, possibly indicative of anticipatory grief (44, 45). Anticipatory grief is feelings of sadness or loss *before* the loss takes place. This occurs when the loss is expected, for example, in the case of an ailing partner. The lower life satisfaction before the loss can also be an indication of a high burden of informal support which has been associated with poorer wellbeing and mental health outcomes in older careers (46). Indeed, the highest proportion of partner loss was found in this trajectory group. This reasoning is also in line with the concept of contextual resilience, as the expectation of impending loss and the new role as caretaker imply a disruption of roles already before the loss (22). After the loss, results suggest gradual adaptation until high levels of life satisfaction are reached again. These results are in line with a study by Infurna et al. (44) showing that individuals with anticipatory declines in life satisfaction prior to partner loss had better adaptation after the loss, potentially indicating the existence of proactive processes that facilitate post-loss adaptation (44). In fact, this is the only trajectory where post-loss levels (means) of life satisfaction are higher than pre-loss levels. This kind of resilient adaptation has been highlighted as it entails growth in the face of adversity (8, 15). In contrast, the “Non-recovering” group shows neither anticipatory grief nor adaptation, but rather a stable decline in life satisfaction starting from the loss. This may be an indication of so-called complicated or prolonged grief (47) in response to partner or functional loss. In complicated grief, there is no adaptation or recovery after the loss, but rather the feelings of distress, sorrow, and anger remain for a long time. The “Non-recovering” group has the highest proportion of childlessness, and over time they decline in perceived access to social support while increasing in reported mental health problems. In the same vein, a recent study found that increased time spent alone after widowhood was the main reason for life satisfaction being lower and not returning to pre-widowhood levels among widowed women (48).

Furthermore, and rather surprisingly, the largest trajectory group was that of a stable linear decline over time without any fluctuations surrounding the loss. This trajectory group also started off with a lower level of life satisfaction compared to the other trajectory groups. Given that they, on average, had more losses during the study period, it may be that the potential accumulation of adversity ultimately led to an inability to adapt and recover, rather than building robustness [e.g., (8, 58]. In addition, the lower initial levels of life satisfaction and poorer mental health, together with the lower education level, may be indicative of various difficulties experienced over the life course prior to our trajectories. In addition, social resources were low in this group—they were more likely to live alone and to not be married/cohabiting before the loss and engaged less with friends and family throughout the study period. Thus, findings add to the literature suggesting that lack of social resources is involved in the non-resilient response to loss [e.g., (22)].

In addition, the importance of social resources is confirmed when looking at the characteristics that predicted resilient trajectory group membership. We found that social resources were indeed central to resilience, in line with earlier studies [e.g., (11, 21, 32)]. Moreover, previous studies have shown that the connections between social interactions and life satisfaction become more robust as individuals advance in age, and their reliance on social networks increases (27). We found that friendships were particularly common in the resilient group. Although the level of engagement with friends was similar across the trajectory groups before the loss, the resilient trajectory groups reported higher friend engagement than the non-resilient groups after the loss. This contrasts with the findings of Netuveli et al. (21), which showed that social support *prior to and during* adversity (measured by functional loss, partner loss or poverty) was most important for resilience in older ages. Nevertheless, the importance of friendships is consistent with studies suggesting that friends have become more important in the lives of older adults (49).

We also included two variables measuring the individuals’ sense of control, namely whether they perceived things that happened as understandable and whether they felt they could influence their lives. These were only measured before the loss. Individuals in the resilient trajectories perceived the world as more understandable and expressed more agency concerning their life situation, similar to the findings of van Kessel (31). This is an interesting finding, which also supports the notion of resilience as an individual trait.

When evaluating the results of this study, several strengths and limitations are important to consider. First, two strengths are that we focus only on older adults and that the study sample is derived from a nationally representative study. Yet the criteria to be included in the analyses produce a potentially select sample and statistical power is limited. Nevertheless, we were able to identify four probable trajectories in the statistical analyses. Second, the long follow-up time adds a unique feature to this study, while potentially also introducing selection bias due to for example mortality and attrition. Because of the generally high response rates in LNU and SWEOLD, we expect the selection bias to be rather limited. Third, the data collection, with its irregular follow-up periods ranging between 2 to 10 years, poses another potential problem. Because the exact timing of the loss is not specified in the data, we have set it in the exact middle of the pre-and post-measurements for everyone. This results in a crude and somewhat diluted measurement of the timing of loss, since it will be either under-or overestimated for most individuals. It may also mean that for some individuals, the long interval between data collections hides potential fluctuations in life satisfaction surrounding the loss that have taken place without being assessed in the data collections. However, since these individuals would likely end up in one of the resilient trajectories, the bias should be rather small. We encourage replication of this study using larger samples with shorter time intervals. Fourth, the statistical method, although allowing more detail and variation than a single mean trajectory, forces all individuals into the trajectory group that they resemble the most. Thus, the trajectories do not represent every individual accurately, and there may be smaller trajectory groups that are not identified. On the other hand, too many trajectory groups may have been extracted due to restricted variances and co-variances in LCGA (25, 41). Fifth, life satisfaction was measured with only one question and not an instrument—which might not be a problem according to a validation study comparing one-item and multiple-item life satisfaction measurements (38), and with slightly different questions and scales across the two data sources, thereby allowing more variation at the later measurement points, despite the rescaling. Finally, we have only included events that happen after age 60 years since our focus is on age-typical events in the older population. Of course, other events than the ones included, both good and bad, and at earlier ages may also influence life satisfaction and resilient adaptation, especially if they accumulate over time. Future studies should investigate these issues further.

In conclusion, population aging has highlighted the need for strengthening and prioritizing individual and societal resources, and one such approach is to focus on individuals’ psychosocial resilience and factors that may help to sustain it in old age (50). Examining resilience can help identify potential protective factors that can be included in interventions at a community level. Confirming earlier results, we found that both social and individual resources are important for resilient responses to loss [e.g., (31)]. Social networks, particularly friendships, a sense of control, and better mental health seem to be central aspects of a resilient adaptation. Policies or actions that increase opportunities for a resilient response to loss and adversity may ultimately enhance the health, independence, and quality of life for many older individuals (2, 51). Our study suggests that interventions that enable older individuals to increase, or at least maintain, their social interactions, particularly following age-related events like the loss of a spouse, may promote resilience in the ageing population.

## Data Availability

The data analyzed in this study is subject to the following licenses/restrictions: Data materials can be made available to other researchers upon reasonable request. University affiliation required. Requests to access these datasets should be directed to www.sweold.se.
