# Supplementary material for: Psychosocial resilience surrounding age-typical losses among older adults in Sweden: group-based trajectories over a 25-year-period
Source: Front Public Health. 2024 Oct 24;12:1434439. doi: 10.3389/fpubh.2024.1434439 (PMC11540683; doi:10.3389/fpubh.2024.1434439)
Supplement: Supplementary file 1 [file Table_1.DOCX]

Supplementary Material

| Table S1. The three models with best BIC | | | | |
| --- | --- | --- | --- | --- |
| Chosen model | Average Posterior Probability | Odds of Correct Classification | Difference between predicted and total probability, % | Entropy |
| Declining: 208 | .93 | 13.8 | .03 | .747 |
| Recovering: 70 | .79 | 18.4 | .02 |  |
| Non-Recovering: 110 | .79 | 13.9 | -.03 |  |
| Bouncing Back: 62 | .88 | 51.1 | -.02 |  |
|  |  |  |  |  |
| Option 2 |  |  |  |  |
| 1. 209 | .92 | 12.5 | .02 | .744 |
| 1. 66 | .82 | 21.1 | .03 |  |
| 1. 113 | .78 | 13.0 | -.04 |  |
| 1. 62 | .87 | 50.7 | -.02 |  |
|  |  |  |  |  |
| Option 3 |  |  |  |  |
| 1. 195 | .88 | 9.2 | .01 | .704 |
| 1. 81 | .81 | 15.0 | .04 |  |
| 1. 116 | .78 | 12.6 | -.03 |  |
| 1. 58 | .87 | 53.5 | -.02 |  |


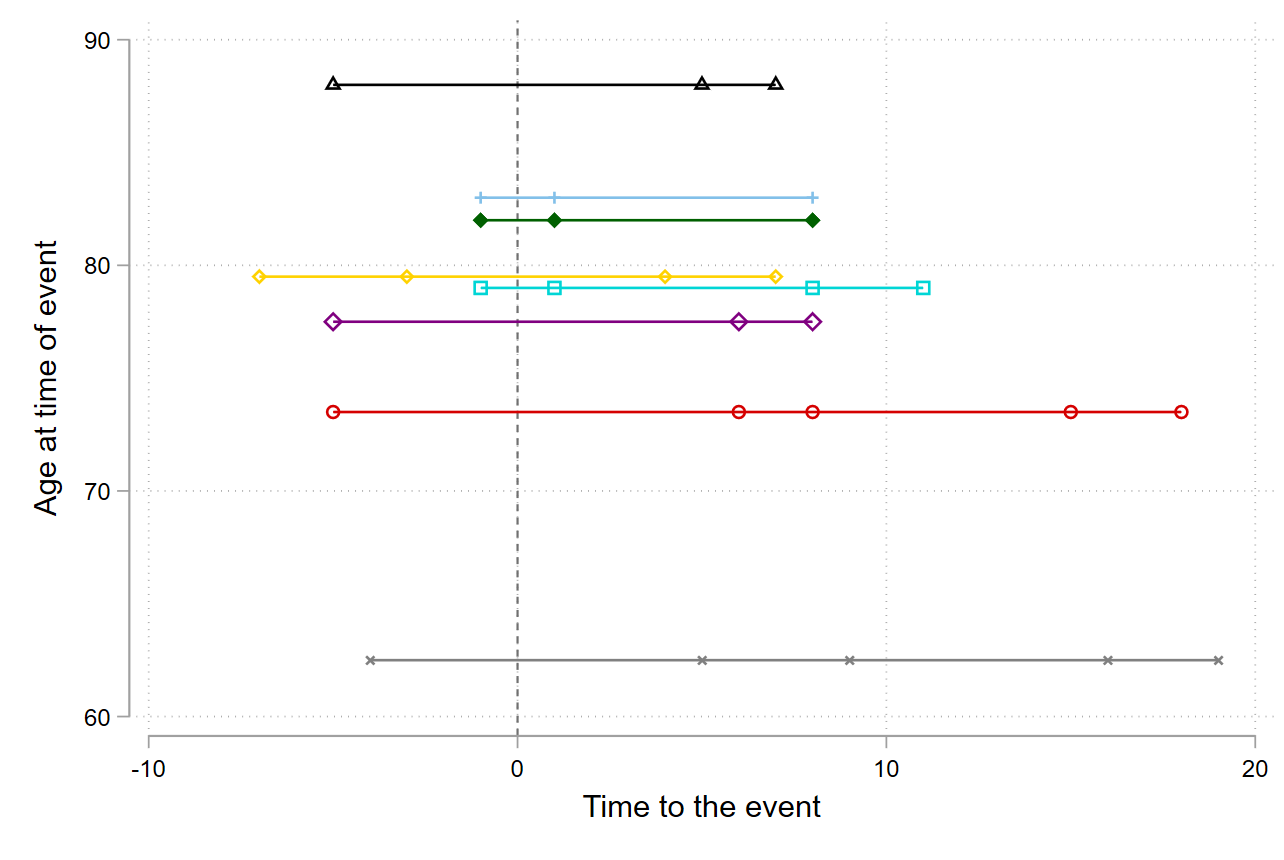


Figure S1. Example of data points from 8 randomly selected participants. Time is measured in years.
